# Postprandial hyperlipidemia, endothelial dysfunction and cardiovascular risk: focus on incretins

**DOI:** 10.1186/1475-2840-10-61

**Published:** 2011-07-07

**Authors:** Sameer Ansar, Juraj Koska, Peter D Reaven

**Affiliations:** 1 Department of Endocrinology, Phoenix Veteran Affairs Healthcare System, 650 E Indian School Rd, CS111E, Phoenix, AZ 85012, USA; 2 Biodesign Institute, Arizona State University, 727 E. Tyler Street Tempe, AZ 85287, USA; 3 Michigan State University, B319 Clinical Center, East Lansing, MI 48824, USA

## Abstract

Cardiovascular disease (CVD) risk in type 2 diabetes (T2DM) is only partially reduced by intensive glycemic control. Diabetic dyslipidemia is suggested to be an additional important contributor to CVD risk in T2DM. Multiple lipid lowering medications effectively reduce fasting LDL cholesterol and triglycerides concentrations and several of them routinely reduce CVD risk. However, in contemporary Western societies the vasculature is commonly exposed to prolonged postprandial hyperlipidemia. Metabolism of these postprandial carbohydrates and lipids yields multiple proatherogenic products. Even a transient increase in these factors may worsen vascular function and induces impaired endothelial dependent vasodilatation, a predictor of atherosclerosis and future cardiovascular events. There is a recent increased appreciation for the role of gut-derived incretin hormones in controlling the postprandial metabolic milieu. Incretin-based medications have been developed and are now used to control postprandial hyperglycemia in T2DM. Recent data indicate that these medications may also have profound effects on postprandial lipid metabolism and may favorably influence several cardiovascular functions. This review discusses (1) the postprandial state with special emphasis on postprandial lipid metabolism and its role in endothelial dysfunction and cardiovascular risk, (2) the ability of incretins to modulate postprandial hyperlipidemia and (3) the potential of incretin-based therapeutic strategies to improve vascular function and reduce CVD risk.

## Review

### Cardiovascular risk in type 2 diabetes

The prevalence of type 2 diabetes mellitus (T2DM) is increasing worldwide at alarming rates. In the next 20 years the prevalence of T2DM is expected to be more than 350 million people worldwide [1]. Cardiovascular disease (CVD) accounts for more than two thirds of all deaths in T2DM [2]. The risk of dying from CVD is nearly twice as high in those with T2DM compared with those of similar age without T2DM and this occurs similarly in both diabetic women and men [3,4]. Consistent with these effects on mortality, T2DM increases the risk of coronary and peripheral artery disease by 2 to 4 fold, while the risk of stroke is increased 10 fold in individuals younger than 55 years of age if they have T2DM [5-7].

Although the risk of major cardiovascular events in diabetes is closely related to glycemic control in observational studies, therapeutic targeting of glycated hemoglobin levels has not been effective in decreasing cardiovascular risk outcomes [8-12]. Furthermore, aggressive management of blood glucose levels per se does not substantially improve most cardiovascular risk factors commonly present in T2DM, including obesity, hypertension or diabetic dyslipidemia indicating that CVD risk management in T2DM requires more than improved glucose control. One of the potential explanations is that most diabetes medications show a neutral, and in some cases even harmful effect on some cardiovascular risk factors. Notably, these risk factors are already present at increased levels in individuals at high risk for T2DM and contribute to their increased cardiovascular risk [13-16].

### Postprandial lipids and cardiovascular risk

Historically, the associations between metabolic abnormalities and cardiovascular disease have been studied largely during fasting conditions. However, the important contribution of postprandial state to cardiovascular disease is increasingly being recognized, particularly in conditions of insulin resistance and T2DM. The mechanisms of postprandial hyperglycemia, as well as its clinical importance (including cardiovascular) risk have been addressed in several excellent review articles [17-20] and we will instead focus on postprandial hyperlipidemia.

Multiple lipid lowering medications have been developed that effectively reduce fasting concentrations of LDL cholesterol and triglycerides (TG). Although several of these medications, particularly the statins, routinely reduce CVD risk by 25-35%, there remains substantial residual and absolute risk in higher CVD risk populations, such as in T2DM. This may be in part explained by postprandial elevation in lipids. In fact, in contemporary post-industrialized societies most individuals spend the majority of non-sleeping hours in the postprandial state. For example, as the typical American diet consists of 3 or more meals per day and it takes more than 8 hours for triglyceride concentrations to return to fasting levels after a meal, postprandial triglyceride concentrations often remain elevated throughout the day. Importantly postprandial triglyceride concentrations may in fact be a better predictor of cardiovascular events than fasting triglycerides. The adverse effect of postprandial triglycerides is thought to be mediated by proatherogenic lipolysis products of nascent triglyceride-rich lipoproteins, such as remnant lipoproteins and fatty acids, and even a transient increase in these factors may worsen vascular function.

Several large observational studies have assessed the association between non-fasting lipid concentrations and cardiovascular risk (Table 1). In the first of these, the association between non-fasting TG concentrations and risk of coronary death was assessed in 37,546 Norwegian male participants, aged 35-49 years, without a history of CVD and diabetes [21,22]. This analysis detected a weak, but statistically significant association between non-fasting TG and coronary death during an average of 9 years of follow-up [22]. When other coronary risk factors were adjusted for, non-fasting TG remained a significant independent predictor of coronary death only in participants within the upper age range, i.e. between 45 and 49 years, and with higher cholesterol levels [22]. In the subsequent analysis performed 4 years later in an even larger cohort of men and women, non-fasting TG were not associated with coronary death in men but showed a 5-fold risk of death from coronary heart disease in women with a non-fasting TG concentration of 3.5 mmol/l or more compared to those with a level of less than 1.5 mmol, even after adjustment for traditional coronary risk factors [21].

**Table 1:** Relative risk of cardiovascular outcomes with non-fasting triglyceride levels

Author (year)	Population	Follow-up	Outcome(s) (number of events)	Adjusted relative risk (95% CI)
Tverdal et al. (1989) [22]	37,546 men aged 35-49 years, without history of CVD or diabetes	9 years (mean)	coronary death (n = 369)	1.1 (1.0-1.2)

Stensvold et al. (1993) [21]	24,535 women, aged 35-49 years, without history of CVD or diabetes	14.6 years (mean)	coronary death (n = 108)	men: 1.1(1.0-1.2) women: 1.6(1.2-2.1)

Stampfer et al. (1996) [23]	14,916 men without history of CVD (85% non-fasting)	7 years	myocardial infarction, cases (n = 266) vs. controls (n = 308)	1.4 (1.1-1.8)

Eberly et al. (2003) [26]	2,809 male participants without clinical evidence of CVD in the MRFIT study	25 years	8-year non-fatal or fatal CHD (n = 175) 25-year fatal CHD (n = 328)	1.6 (1.2-2.3) fasting 1.5 (1.0-2.1) non-fasting 1.2 (1.0-1.6) fasting 1.3 (1.0-1.6) non-fasting

Nordestgaard et al. (2007) [24]	7,587 women and 6,394 men form the general population in Copenhagen (Denmark)	26 years (mean)	myocardial infarction (n = 1,793) ischemic heart disease (n = 3,479)	1.2 (1.1-1.4) women 1.0 (1.0-1.1) men 1.1 (1.0-1.2) women 1.0 (1.0-1.1) men

Bansal et al. (2007) [27]	26,509 healthy US women, 20,118 fasting, 6,391 non-fasting (<8 hours since last meal)	11.4 years (mean)	cardiovascular events (n = 1001)	1.1 (0.9-1.3) fasting 1.7 (1.2-2.4) non-fasting 4.5 (2.0-10.2) 2-4 hrs since last meal

The Physicians' Health Study, a prospective nested case control study was conducted in 14,916 men aged 40 to 84 years, 85% of whom had baseline blood samples taken under non-fasting conditions [23]. The primary outcome was occurrence of myocardial infarction (MI) during 7 years of follow-up. A key finding was that cases (n = 266) had higher median non-fasting TG levels compared to controls (n = 308). After simultaneous adjustment for age, smoking status, HDL- and total cholesterol levels, LDL diameter and a variety of coronary risk factors, non-fasting TG concentrations significantly predicted future risk of MI.

Non-fasting TG were also associated with myocardial infarction (MI) and ischemic heart disease and death after 26 years of follow-up in a prospective cohort of almost 14,000 women and men in the Copenhagen study [24]. The association was strongest among the individuals with higher categories of non-fasting triglyceride concentrations, but was weaker when TG levels were examined as a continuous variable and predicted MI in fully adjusted multivariate models only in women. In an additional analysis of this cohort, cumulative incidence of ischemic stroke was also directly proportional to the levels of the non-fasting TG [25].

Since none of the above studies included measurement of fasting lipids, it is not clear whether similar relationships with CVD would be observed with fasting TG values. However, the combination of a screening visit without requirements to be in fasting condition along with a protocol for strictly fasting enrollment visits in 2,809 asymptomatic men who participated in the Multiple Risk Factor Intervention Trial provided an opportunity to compare the effects of both fasting and non-fasting TG levels on non-fatal and fatal coronary heart disease [26]. The analyses showed a mild association of TG with CVD, which was similar for both fasting and non-fasting TG after 8 years of follow-up for both fatal as well as non-fatal coronary heart disease (CHD) events and after 25 years of observation for fatal CHD events [26]. A limitation of the study was that the time since the last meal prior to the non-fasting visit blood draw was not recorded.

In contrast, based on the time after the last meal for baseline blood draws, 26,509 initially healthy women in Women's Health Study [27] were stratified into fasting (8 and more hours since last meal) and non-fasting groups (meal within 8 hours prior to blood collection). After adjustment for standard cardiovascular risk factors including total and HDL cholesterol, diabetes status, body mass index (BMI) and C-reactive protein (CRP), fasting TG levels were not associated with incident cardiovascular events over an 11-year follow-up period. On the other hand, non-fasting triglycerides maintained a strong independent relationship with future cardiovascular events even in the fully adjusted analyses. Moreover, after stratification by time since the last meal, TG concentrations measured 2-4 hours after the last meal were the strongest predictor of CVD events.

### Postprandial lipid metabolism and vascular risk

Triglycerides of dietary origin enter the lymph and then systemic circulation as chylomicrons packaged together with apolipoprotein B-48 (apoB-48). Following a fat enriched meal, chylomicrons rapidly increase and lead to pronounced triglyceride elevations peaking approximately 4 hours post-meal. In humans, the presence of apoB-48 distinguishes chylomicrons from smaller VLDL particles carrying triglycerides of hepatic origin that contain apolipoprotein B-100 (apoB-100). Although increases in particles carrying apoB-48 explain about 80% of the postprandial triglyceride increase, much of the increase in the particle count is represented by particles containing apoB-100 [28]. Both chylomicrons and VLDL lipoproteins are cleared from the circulation after undergoing lipolysis by several lipases, i.e. lipoprotein or hepatic lipases, producing fatty acids and smaller remnant lipoprotein particles (RLP). Since these enzyme pathways have limited capacity, there is competition in clearance between both VLDL and chylomicrons [28]. One important consequence of chylomicrons lipolysis is that it reduces the size of lipoproteins sufficiently to permit entry into the arterial wall, where proatherogenic properties of these modified lipoproteins (described below) may alter vascular function.

RLPs after lipolysis become enriched in cholesterol, deposit 5- to 20-fold more cholesterol into the vessel wall per particle, and are preferentially retained in the vessel wall where they are avidly taken up by macrophages [29-31]. RLPs have been shown to be proinflammatory causing endothelial dysfunction, and induction of monocytes chemoattracting protein 1 (MCP-1) expression by vascular smooth muscle cells [32,33]. Consistent with this, increased RLPs have also been associated with coronary artery disease, and predict progression of atherosclerosis and development of cardiovascular events [34-36]. Postprandial changes in RLPs closely correlate with postprandial changes in TG [37]. Apolipoprotein CIII (apoCIII) appears to be an important inhibitor of RLPs clearance [38,39]. Furthermore, recent *in vitro *and *in vivo *experimental data showed that apoCIII may directly induce endothelial dysfunction via inhibition of insulin-induced activation of Akt resulting in reduced nitric oxide (NO) release and subsequent impaired vasodilation [40]. Possibly as a consequence, high levels of apoCIII in plasma, or in VLDL particles, are associated with increased risk of CVD [39,41].

Fatty acids, an additional product of postprandial TG hydrolysis, may also contribute to increased cardiovascular risk. Increased non-esterified fatty acids (NEFA) concentrations are prospectively associated with both cardiovascular morbidity and mortality [42,43]. NEFA effects on the cardiovascular system include injury to both the myocardium and increased susceptibility to arrhythmias [44], and to the vasculature, by stimulation of inflammatory processes, local production of reactive oxygen species and impaired endothelial dependent vasodilation [45-47]. Fatty acids could further facilitate development of atherosclerotic plaque through stimulation of inflammatory processes in macrophages [48].

### The postprandial state and endothelial dysfunction

Endothelial dysfunction is believed an important link between the postprandial state, atherosclerosis and CVD. It is characterized by impaired endothelium-dependent vasodilation and increased pro-coagulant and pro-inflammatory activity [49]. Coronary endothelial dysfunction has been shown to predict cardiovascular events in patients with and without coronary artery disease [50-52]. Although coronary endothelial dysfunction is commonly present in individuals with a long history of T2DM, it is also present in those with insulin resistance, prediabetes and/or new onset T2DM [53,54]. Studies have demonstrated that endothelial function in both healthy subjects and those with T2DM is altered by meals with high amounts of fat or carbohydrates [55,56] and is inversely related to both glucose and TG concentrations [56-58]. Since endothelial dysfunction is a diffuse process, measurement of endothelial function in peripheral arteries can be used as a surrogate of coronary endothelial function. Peripheral endothelial function correlates well with coronary endothelial vasodilation and is reduced in patients with cardiovascular risk factors such as obesity, hypercholesterolemia, hypertension and diabetes [54,59-62]. Lower peripheral endothelial function also predicts progression of carotid atherosclerosis in the general population as well as cardiovascular morbidity and mortality in populations with high CVD risk [63-68].

### Endogenous incretin hormones in regulation of glucose, lipid and vascular responses

**Figure 1:**
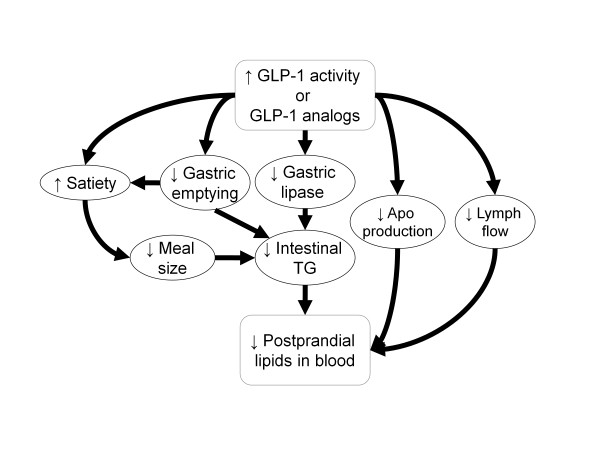
**A scheme of the complex effect of incretin activity on postprandial lipids**. GLP-1 or GLP-1 receptor agonists acting on the central nervous system increases satiety and therefore reduces nutrient intake. Inhibitory GLP-1 activity on gastric emptying both further increases satiety and slows entry of nutrients including lipids into the intestine. Triglyceride (TG) absorption into intestinal cells is further reduced because of incretin-induced inhibition of gastric lipase. In the intestinal cells, incretins also decrease production of apolipoproteins (Apo) B-48 and A-IV thereby inhibiting intestinal biosynthesis of triglycerides and their secretion into blood. Transport of lipids from intestinal cells to blood may be further reduced by inhibitory effect of incretins on intestinal lymph flow. This combination of effects leads to lowering of postprandial lipid levels in blood.

The incretin hormones, glucagon-like peptide-1 (GLP-1) produced by L-cells in the distal gut, and glucose-dependent insulinotropic polypeptide (GIP) produced by duodenal K-cells in response to ingested nutrients, are important regulators of glucose homeostasis [69]. Incretins are secreted into the circulation within minutes in response to a meal and upon release they bind to specific G-protein coupled receptors present on β-cells and other target tissues [70,71]. GLP-1 is secreted in greater concentrations than GIP and is considered more physiologically relevant in humans [72]. In β-cells, GLP-1 enhances glucose-dependent insulin secretion, increases insulin synthesis, and in animals stimulates β-cell proliferation and inhibits apoptosis [69]. GLP-1 also reduces glucose concentrations through inhibition of pancreatic α-cell glucagon secretion and indirectly via inhibition of gastric emptying and appetite [73-75]. Importantly, in addition to slowing gastric emptying, GLP-1 may also decrease intestinal lymph flow, triglyceride absorption, and apolipoprotein synthesis adding to a complex combination of mechanisms that may limit the release of triglycerides into the circulation after lipid-containing meals [76] (Figure 1). Consistent with this, administration of GLP-1 or GLP-1 receptor agonists in humans is associated with significant reduction of postprandial lipids (Table 2). Intravenous infusion of GLP-1 abolished the rise in postprandial triglyceride concentrations in healthy men [77]. Furthermore, it also decreased fasting and postprandial NEFA concentrations [77] in agreement with a previous report on the effect of 6-week continuous subcutaneous GLP-1 infusion in patients with T2DM [78] (Table 2).

**Table 2:** The effect of incretins or incretin based therapies on postprandial lipid metabolism in humans

Compound	Author	Intervention	Design	Study population	Findings
GLP-1	Meier et al. (2006) [77]	390-min IV infusion	randomized, double blinded, placebo-controlled crossover study	14 healthy male volunteers	Reduced postprandial triglyceride and NEFA levels

GLP-1	Zander et al. (2002) [78]	6-week continuous SQ infusion	randomized, single-blinded, placebo controlled parallel study	20 patients with T2DM (10 in each group)	Decreased fasting and average 8-h post-meal NEFA levels

Exenatide	Cervera et al. (2008) [98]	6-hour continuous IV infusion	non-randomized single-blinded crossover study vs. control	12 subjects with T2DM	Reduced triglyceride response to mixed meal

Exenatide	Schwartz et al. (2008) [99]	2-week SQ injection twice a day	randomized, double-blinded, placebo-controlled parallel study	30 patients with inadequately controlled T2DM	Decreased morning and evening postprandial triglyceride excursions, no effect after midday meal

Exenatide	Schwartz et al. (2010) [103]	Single SQ dose just before a high-fat meal	randomized, double-blinded, placebo-controlled crossover study	35 patients with impaired glucose tolerance or recent T2DM	Abolished responses of triglyceride, NEFA, RLPs, apoB48 and apoCIII to meal

Exenatide or Sitagliptin	DeFronzo et al. (2008) [100]	2-week SQ exenatide injection twice a day or sitagliptin orally once/day	double-blinded randomized crossover study	61 patients with T2DM treated with a stable regimen metformin	Reduced average 4-h post-meal triglyceride response after both. Reduction greater after exenatide (by ~10%)

Vildagliptin	Matikainen et al. (2006) [107]	4-week oral dose 50 mg twice/day	double-blinded randomized placebo-controlled parallel study	31 drug-naïve T2DM patients (n = 16 allocated to Vildagliptin)	Decreased postprandial TG-rich lipoproteins (total and chylomicrons, apoB-48)

SQ, subcutaneous; IV, intravenous;

Notably, the incretin effect appears reduced or lost in individuals with impaired glucose tolerance and T2DM [79,80]. This is most commonly ascribed to reduced endogenous levels of GLP-1. However, as GLP-1 receptor signaling remains intact, continuous administration of GLP-1 effectively reduces blood glucose levels in patients with T2DM [81,82].

Recent studies indicate that GLP-1 may also exert beneficial effects on the cardiovascular system independent of its effects on glucose, lipid or energy metabolism [83]. *In vitro*, the active form of GLP-1 (7-36) induced endothelium-dependent vasodilation in preconstricted pulmonary arteries [84]. *In vitro*, GLP-1 inhibited tumor necrosis factor alpha (TNF-α) induced plasminogen activator inhibitor 1 (PAI-1) gene and protein expression in endothelial cells [85]. *In vivo*, administration of GLP-1 improved endothelial function in salt-sensitive hypertensive rats [86]. Of great relevance, pharmacological levels of GLP-1 improved endothelial function in healthy individuals as well as in T2DM patients with stable coronary artery disease [87,88] and had a protective effect on postprandial endothelial function [89]. In addition to these vascular effects, GLP-1 or GLP-1 receptor agonists demonstrated multiple beneficial actions on the heart including protection of myocardium from ischemia in rats [90], improvement of cardiac function in rats with congestive heart failure [91] and attenuation of ischemic left ventricular dysfunction during stress echocardiography in patients with coronary artery disease [92].

### Incretin-based therapies to reduce postprandial dyslipidemia and improve endothelial dysfunction

Although GLP-1 is highly effective in lowering blood glucose and has promising cardiovascular effects, its therapeutic potential is severely limited because of rapid degradation by dipeptidyl peptidase 4 (DPP-4) to GLP-1 (9-36) which does not stimulate GLP-1 receptor and therefore does not exert the metabolic effects of active GLP-1 [93]. Therefore, the therapeutic focus has been directed towards compounds that either mimic the activities of GLP-1 while being less susceptible to degradation by DPP-4 or that reduce degradation of endogenous GLP-1 (and GIP) by inhibiting DPP-4. However, the effect of these therapies on CVD is unknown.

Exenatide (exendin-4), the first FDA approved GLP-1 mimetic, has only 53% homology to the human GLP-1 amino acid sequence; as such, it is relatively more resistant to DPP-4, reaching maximum levels approximately 2 hours following subcutaneous injection [94]. Exenatide reproduces many of the action of GLP-1, such as enhancement of glucose-induced insulin secretion, inhibition of glucagon release, reduction of fasting and postprandial glucose concentrations, delay of gastric emptying, inhibition of appetite and induction of weight loss [94-97]. Acute infusion or short-term treatment with exenatide twice a day abolished increments in postprandial triglyceride concentrations in patients with T2DM [98-100] (Table 2). Long-term treatment with exenatide was associated with significant improvement in multiple cardiovascular risk factors including systolic and diastolic blood pressure, fasting triglycerides, as well as total, LDL- and HDL-cholesterol [101]. Recent experimental data indicate anti-atherosclerotic effects of exenatide involving inhibition of inflammatory responses of atherosclerotic plaque macrophages [102].

**Figure 2:**
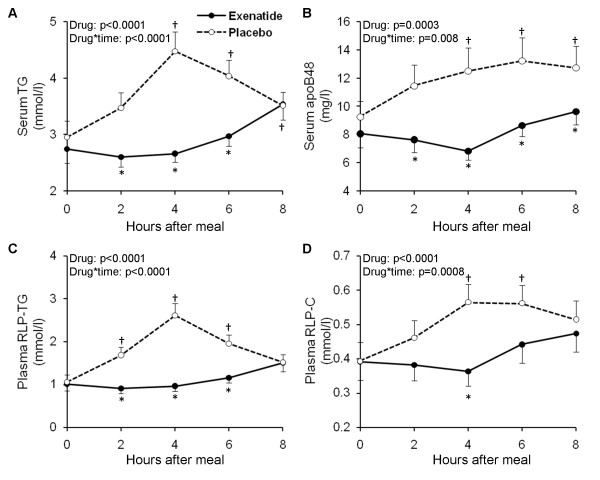
**The effect of exenatide or placebo on postprandial concentrations of triglycerides (panel A) and apolipoprotein B-48 (apoB48, panel B) in serum, and remnant lipoprotein triglycerides (RLP-TG, panel C) and cholesterol (RLP-C, panel D) in plasma**. The average effect of study medication (Drug) and the interaction between the effects of meal and drug (Drug*Time) were evaluated by repeated measures ANCOVA (adjusted for test sequence and glucose tolerance status). Symbols denote statistically significant (p < 0.05) difference between exenatide and placebo (‡) and versus pre-meal value (*) at each specified time points tested by post-hoc multiple comparison analyses. Number of subjects included in the analyses: triglycerides, n = 35; RLP-TG, n = 34; RLP-C, n = 31; apoB48, n = 28 (Schwartz et al., *Atherosclerosis *2010, **212**(1):217-222 [103]).

To assess the effects of typical clinical dosing of exenatide on postprandial lipid and lipoprotein excursions, we conducted a double-blinded, randomized, placebo-controlled, crossover study in participants with IGT or with recent onset T2DM in good control with diet alone [103]. The intervention was a single subcutaneous injection of exenatide (10 μg) or normal saline just prior to a high-caloric (600 kcal/m^2^), fat-enriched breakfast meal (45% fat, 40% carbohydrates, 15% proteins). Blood was collected for lipid assays over an 8 hour postprandial period. The single dose of exenatide strongly suppressed postprandial elevation of TG, apoB-48, RLP-TG and RLP-cholesterol (Figure 2) as well as apoCIII, whereas declines in NEFA were less pronounced but persisted longer in exenatide compared with placebo (Table 2). These effects of exenatide were similar in those with IGT or recent onset T2DM demonstrating that exenatide profoundly inhibits postprandial excursions of proatherogenic lipids and lipoproteins and may reduce cardiovascular risk in those early in the evolution of diabetes. These lipid and lipoprotein lowering effects of exenatide were present regardless of concomitant therapy of dyslipidemia with statins.

**Figure 3:**
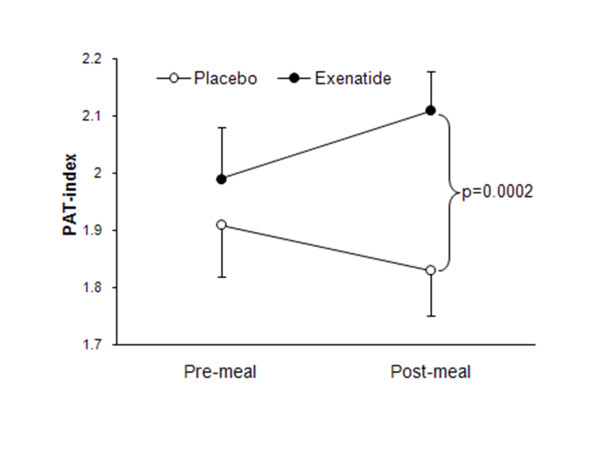
**The effects of exenatide and placebo on postprandial endothelial function**. Endothelial function was measured before and after a single high-fat breakfast meal. Participants received placebo and exenatide on separate visits in a cross-over design. Post-meal PAT index was significantly higher (demonstrating improved endothelial function) during the exenatide phase compared with the placebo phase (p = 0.0002, adjusted for pre-meal PAT index, treatment sequence and glucose tolerance status) (Koska et al., *Diabetes Care *2010, **33**(5):1028-1030 [104]).

We also tested whether the metabolic effects of exenatide would translate to improved postprandial endothelial dysfunction [104]. Twenty eight of the study participants successfully completed both pre- and post-meal measurement of peripheral endothelial function using finger plethysmography (peripheral arterial tonometry - PAT). A single exenatide administration was followed by significant improvement of postprandial endothelial function compared to the placebo treatment (Figure 3). Two thirds of exenatide's effect was explained by changes in triglyceride concentrations indicating that modulation of postprandial lipid metabolism played a major role in observed improvement of endothelial function. In agreement with previous reports of postprandial endothelial dysfunction in individuals with T2DM [56], our data also reinforced increased susceptibility of diabetic individuals to postprandial impairment of endothelial function. Furthermore, they suggested that this susceptibility develops very early after onset of T2DM as it was absent in persons with IGT and present in the recently diagnosed T2DM subjects with good glycemic control. Importantly, the benefit of exenatide on postprandial endothelial function was similar among those with IGT and diabetes, i.e. it prevented the postprandial decrease of endothelial function in T2DM and induced an increase in endothelial vasodilation of similar magnitude in IGT, demonstrating that exenatide therapy beneficially influences endothelial function in both prediabetes and early stages of T2DM.

Clinical studies with DPP-4 inhibitors provide additional support for incretin-based therapies as an effective tool in ameliorating postprandial dyslipidemia. These compounds only moderately increase endogenous GLP-1 levels and as such they do not have a detectable effect on gastric emptying rate [105,106]. Despite this, the ability of the DPP-4 inhibitor sitagliptin to reduce postprandial TG levels was only about 10% less than exenatide in a two-week cross-over study in patients with T2DM [100] (Table 2). In a separate double-blinded placebo-controlled study in drug-naïve T2DM patients, Matikainen et al. [107] demonstrated that 4-weeks therapy with another DPP-4 inhibitor, vildagliptin, also decreased postprandial TG levels (Table 2). They also demonstrated that the reduction in triglycerides was accounted for by reduced TG in chylomicrons and lower apoB-48. In contrast, there were not significant changes in VLDL TG or apoB-100 levels [107]. A direct inhibitory effect of incretins on intestinal lipid secretion is further supported by data showing that intestinal production of apoB-48 both *in vitro *and *in vivo *in rodents was profoundly reduced by both exendin-4 and sitagliptin [108]. A summary of these incretin-mediated mechanisms contributing to lowering of postprandial lipids is illustrated in Figure 1.

### Prospects for incretin-based therapies to reduce cardiovascular risk

Since more intensive glycemic control with standard diabetes medications has been largely ineffective in reducing the high residual cardiovascular risk in individuals with T2DM, aggressive treatment of other cardiovascular risk factors appears to be the logical next step in the effort to decrease cardiovascular risk in this group. In fact, the results of the STENO-2 study show that a target-driven, long-term, intensified intervention aimed at multiple risk factors in patients with type 2 diabetes and microalbuminuria, may reduce the risk of cardiovascular events by about 50 percent [109]. An increasing body of clinical evidence indicates that incretin-based therapies not only lower fasting and postprandial glucose, but also improve a wide variety of traditional cardiovascular risk factors including obesity, high blood pressure as well as fasting and postprandial lipid and apolipoprotein concentrations [99-101,107,110-113]. Importantly, the effect of incretins on postprandial lipid excursions appears acute and therefore may be additive to lipid benefits of decreased appetite and body weight reduction that are characteristic of more chronic therapy with incretins or incretin analogues [77,98,103]. Moreover, some experimental data in humans show that increased GLP-1 activity may directly stimulate endothelial-mediated vasodilation independently of known metabolic actions of GLP-1 [87,88,104]. Exendin-4 also attenuated atherosclerotic lesions in mice model of atherosclerosis [102]. The attenuation was associated with reduced monocyte/macrophage accumulation in the arterial wall indicating that suppression of vascular inflammation may represent another direct cardiovascular benefit of incretin-based therapy [102].

## Conclusions

Although this review highlights clinical and experimental data that provide evidence for favorable cardiovascular effects of incretins, the lack of outcome studies and the short history of clinical use of these agents limit our knowledge about their clinical cardiovascular efficacy. Large multi-center longitudinal studies designed not just to prove CVD safety as shown in two recent meta-analyses of short-term randomized clinical trials of exenatide [114] or sitagliptin [115], but to demonstrate cardiovascular benefits of incretin-based strategies, appear warranted and have recently been initiated (Table 3). In support of this goal, post-hoc analyses within the ACCORD cohort indicated that the only diabetes medication associated with a decrease in CVD events was exenatide [116]. Furthermore, a retrospective study using LifeLinkTM database of medical and pharmaceutical insurance claims for June, 2005 through March, 2009 found that 39,275 patients with type 2 diabetes were treated with exenatide twice daily were less likely to have a CVD event, lower rates of CVD-related and all-cause hospitalization compared to 381,218 patients treated with other glucose-lowering therapies [117]. Moreover, mathematical models accounting for the constellation of CVD risk factors affected by incretin analogs (exenatide in this specific case) predict greater reductions in major adverse cardiovascular events than glucose-lowering regimens without incretins [118]. However, the results of ongoing studies will clarify whether incretin-based therapies live up to their promise as vasculoprotective agents.

**Table 3:** Recently launched multi-center, randomized, placebo controlled longitudinal studies evaluating cardiovascular benefit of incretin-based therapies in individuals with type 2 diabetes (Source: http://www.clinicaltrials.gov).

Trial	Active drug	Category	Phase	N	Start	Duration
**EXSCEL**	Exenatide QW (weekly)	GLP-1 agonist	III	9,500	June 2010	6 3/4 years

**LEADER**	Liraglutide	GLP-1 agonist	IV	8,754	August 2010	6 1/4 years

**TECOS**	Sitagliptin	DPP-IV inhibitor	IV	14,000	December 2008	6 years

**SAVOR-TIMI53**	Saxagliptin	DPP-IV inhibitor	IV	12,000	May 2010	5 years

## List of abbreviations

apoB-100: apolipoprotein B-100; apoB-48: apolipoprotein B-48; apoCIII: apolipoprotein CIII; BMI: body mass index; CHD: coronary heart disease; CVD: cardiovascular disease; CRP: C-reactive protein; DPP-4: dipeptidyl peptidase 4; GIP: glucose-dependent insulinotropic polypeptide; GLP-1: glucagon-like peptide-1; IGT: impaired glucose tolerance; MCP-1: monocytes chemoattracting protein 1; MI: myocardial infarction; NEFA: non-esterified fatty acids; NO: nitric oxide; PAI-1: plasminogen activator inhibitor 1; PAT: peripheral arterial tonometry; RLPs: remnant lipoprotein particles; T2DM: type 2 diabetes mellitus; TNF-α: tumor necrosis factor alpha.

## Competing interests

JK and PDR are recipients of unrestricted research grants from Amylin/Lilly. SA declares no competing interests.

## Authors' contributions

SA and JK drafted the manuscript. PDR conceived of the concept, initiated and helped drafting, and reviewed the final version of the manuscript. All authors read and approved the final manuscript.
